# Abnormal Uterine Bleeding In Adolescents

**DOI:** 10.4274/jcrpe.0014

**Published:** 2018-07-31

**Authors:** Selin Elmaoğulları, Zehra Aycan

**Affiliations:** 1 University of Health Sciences, Dr. Sami Ulus Obstetrics and Gynecology, Pediatric Health and Disease Training and Research Hospital, Clinic of Pediatric Endocrinology, Ankara, Turkey; 2 University of Health Sciences, Dr. Sami Ulus Obstetrics and Gynecology, Pediatric Health and Disease Training and Research Hospital, Clinic of Pediatric Endocrinology and Clinic of Adolescent Medicine, Ankara, Turkey

**Keywords:** Abnormal uterine bleeding, heavy menstrual bleeding, adolescents

## Abstract

Abnormal uterine bleeding (AUB) is the most common gynecologic complaint of adolescents admitted to hospital. Heavy menstrual bleeding (HMB) is the most frequent clinical presentation of AUB. Anovulatory cycles, owing to immature hypothalamic-pituitary-ovarian axis, is the leading etiology of HMB and there is an accompanying bleeding disorder in almost 20% of patients with HMB. Additionally, endocrine disorders such as hypothyroidism, hyperprolactinemia and polycystic ovary syndrome are possible causes of AUB. Exclusion of bleeding disorders, especially of von Willebrand disease is important for diagnosis and treatment of HMB, particularly in cases with AUB, which has been present since menarche. Management of HMB is based on the underlying etiology and severity of the bleeding. After other causes are excluded, anovulatory heavy bleeding can be treated successfully with combined oral contraceptives and iron supplementation either as an outpatient or in hospital depending on the clinical findings and level of anemia. The epidemiology, clinical presentation, diagnostic approach and treatment of HMB is discussed and our clinical experience in this field is presented in this review.

## Introduction

Abnormal uterine bleeding (AUB) is defined as bleeding from the uterine corpus that is abnormal in duration, volume, frequency and/or regularity. AUB accounts for half of the gynecologic problems among adolescents (1,2). Also, some adolescents maybe unaware that their bleedings patterns are abnormal, as menstrual cycles are known to often be irregular during adolescence. The underlying factors that cause AUB and/or AUB itself may have potential for long term health consequences, decrease life quality and affect school attendance. Evaluation of the menstrual cycle should be an additional vital sign to be looked into in any female adolescent during all routine pediatrician visits (3). Beginning with definition of normal menstrual cycle, evaluation, approach to diagnosis and treatment of AUB will be discussed in this paper.

## Normal Menstrual Cycles in Adolescents

Menarche usually occurs between the ages of 12-13 years (4,5). The normal cycle of an adolescent female occurs every 21-45 days with bleeding lasting between two and seven days (6,7,8). The frequency of cycles decreases at higher postmenarchal ages (7). Menstrual cycles are 21-34 days, similar to adults, in 60-80% of adolescents by the third year after menarche (8,9). The average blood loss during a normal menstrual cycle is 30-40 mL, requiring the use of 3-6 pads or tampons per day or 10-15 soaked pads or tampons per cycle (10). More than 50% of the total menstrual loss is an endometrial transudate and 30-50% consists of whole blood components (11). Chronic loss of ≥80 mL blood is associated with anemia (12).

## Abnormal Uterine Bleeding

The International Federation of Gynecology and Obstetrics (FIGO) recommends the use of the term AUB to describe any aberration of menstrual volume, regulation, duration and/or frequency in a woman who is not pregnant (Table 1). FIGO also proposes to discard some definitions from accepted terminology, such as “menorrhagia”, “metrorrhagia”, “hyper/hypomenorrhea”, “polymenorrhea” and “dysfunctional uterine bleeding” as they are controversial, confusing and poorly defined (13,14).

Heavy menstrual bleeding (HMB) is the most common clinical presentation of AUB. Formerly called “dysfunctional uterine bleeding”, refers to AUB which is not caused by structural lesions of the uterus (15).

FIGO defines the etiology of AUB using the PALM-COEIN classification [Polyp, Adenomyosis, Leiomyoma, Malignancy, Hyperplasia (structural causes); Coagulopathy, Ovulatory dysfunction, Endometrial, Iatrogenic and Not yet classified (non-structural causes)] system (1). AUB is very rarely due to structural problems (1.3-1.7%) in adolescents (16,17). Anovulatory cycles, discussed separately below, which may manifest as amenorrhea, oligomenorrhea or HMB owing to immature hypothalamic-pituitary-ovarian axis are the most common cause of AUB among adolescents (18). As another leading etiology, coagulopathy prevalence is reported to vary between 5% and 28% among hospitalized adolescents with HMB in different studies (17,19,20,21,22). In a systematic review gathering data of 988 women (15-55 years) with HMB, the incidence of von Willebrand disease (vWD) was found to be 13% (23). Coagulopathy may also be due to other coagulation factor deficiencies, immune thrombocytopenia, platelet dysfunction, thrombocytopenia secondary to malignancy or due to treatments for malignancy (17,22,24,25). Coagulopathy may be an isolated or accompanying disorder. More than one cause may exacerbate or aggravate AUB. The differential diagnosis of HMB in adolescents is summarized in Table 2 (26).

## Anovulatory Cycles

Occurrence of ovulatory menstrual cycles require the regular interaction of hypothalamus, hypophysis, ovary and endometrium. Gonadotropin releasing hormone (GnRH) pulses from the hypothalamus induce follicle stimulating hormone (FSH) and luteinizing hormone (LH) secretion from the hypophysis and these gonadotropins induce the development of a dominant follicle from one of the antral follicle candidates for ovulation. LH stimulates the thecal cells to divide and produce androgens. FSH stimulates the granulosa cells to divide and convert androgens to estradiol (E2) and E2 level continue to rise through the follicular phase. When E2 exceeds a critical level (>200 pg/mL for two days) GnRH rises with positive feedback and causes an LH surge. This LH surge activates proteolytic enzymes which leads to follicular rupture and causes luteinization of the granulosa and theca cells, resulting in a marked increase in progesterone production.

E2 induces endometrial epithelial cell proliferation, gland growth and vascularization and production of both E2 and progesterone receptors thus preparing the endometrium to respond to luteal production of progesterone. Progesterone stabilizes the thickening endometrium by inﬂuencing the production of key proteins such as matrix metalloproteinase 1, 3, and 9 which degrade extravascular and stromal matrix (27). Progesterone also stimulates production of tissue factor and plasminogen activator inhibitor 1, expediting coagulation and clot stabilization (28,29).

Pituitary potential to respond to GnRH stimulation and the positive feed-back effect of E2 progressively improve after menarche (30,31). During the first two postmenarchal years, approximately half of menstrual cycles are anovulatory. However, at five years post-menarche 75% of cycles are ovulatory and this increases further over the next several years, reaching an 80% rate (31). Delayed or absent ovulation, either physiological or due to polycystic ovary syndrome (PCOS), results in lack of progesterone and excessive E2 production from ovarian follicles, causing the endometrium to proliferate and to become prone to unpredictable menstrual bleeding in both timing and amount. For these reasons anovulatory cycles are the leading cause of HMB during adolescence.

## Evaluation

The focus of initial evaluation of a patient with HMB is to determine whether the bleeding is acute and causing hemodynamic instability, through careful history taking, physical examination, laboratory testing and radiologic imaging.

History should be taken both with and without the parents being present because some of the questions asked would be difficult for patients to answer candidly in the presence of their parents, especially those relating to sexual activity, while asking with the parents present may help to clarify the details in some cases. History should include; menstrual history (age of menarche, regularity, duration, number of pads/tampons per day), sexual history, past medical history (systemic illness, current/recent medication), systemic review (symptoms associated with systemic causes of HMB such as obesity, PCOS, hypothyroidism, hyperprolactinemia, hypothalamic or adrenal disorder) and family history (coagulopathy, hormone sensitive cancers). A history of heavy menses since menarche, surgery related bleeding, bleeding associated with dental work, bruising or epistaxis with a frequency of at least once per month, frequent gum bleeding and bleeding symptoms in the family point to an underlying bleeding disorder (32).

Once hemodynamic stability is established, vital signs should be checked and systematic physical examination should be completed. Presence of goiter, pallor, bruising, petechiae and/or signs of androgen excess may clarify the underlying diagnosis (33). Pelvic examination with a speculum or transvaginal ultrasonography may not be possible in sexually inexperienced adolescents. It is possible to postpone this exam until a trial of medical therapy has been attempted, as structural lesions in adolescents are very rare. Pelvic ultrasonography provides non-invasive information about genital tract structural lesions, especially in adolescents in whom the physical examination is limited. It also gives additional information about endometrial thickness and PCOS.

Laboratory tests are aimed at determining the severity of the bleeding and to investigate potential etiologies of HMB. The minimum laboratory evaluation should include; human chorionic gonadotropin, complete blood count, peripheral blood smear, ferritin level, prothrombin time, activated partial thromboplastin time and fibrinogen. Adolescents at risk of bleeding disorders should undergo testing for vWD. The von Willebrand panel should include; plasma von Willebrand factor (vWF) antigen and functional tests for vWF and factor VIII activity (32,34). Those with a blood type O will have lower levels of vWF than those who have blood type A or B. So, vWF antigen reference values should be used which are appropriate for each patient’s blood type (35). The Committee on Adolescent Health Care of the American College of Obstetricians and Gynecologists recommends obtaining a vWF panel either before or seven days after ceasing exogenous estrogen treatment (36). Estrogen replacement therapy has been shown to increase plasma vWF antigen (37) thus the rationale for the timing of the vWF panel which allows the levels to stabilize in respect to any medical estrogen therapy which may be being used. Additional tests include exclusion of infection in sexually active adolescents and evaluation of thyroid functions in patients with accompanying hypothyroid symptoms. Patients with a significant bleeding history and non-diagnostic initial testing should be referred to a hematologist for further investigation (38).

## Treatment

Providing hemodynamic stability, correction of anemia and maintenance of normal cycles constitute the main goals in management of HMB. Treatment options include iron supplementation, combined oral contraceptives (COCs), progesterone, nonsteroidal anti-inflammatory drugs (NSAIDs), antifibrinolytics, desmopressin and GnRH analogues. Management is largely based on severity of the bleeding and anemia (39). If an underlying cause is identified, specific treatment is given additionally. As HMB in adolescents is mostly due to anovulatory cycles, treatment is focused on anovulatory uterine bleeding. Classification of severity is given in Table 3.

**Mild Anovulatory Uterine Bleeding: **For girls with mild bleeding with normal hemoglobin, observation is enough, unless they report a negative change in their life quality. NSAIDs, such as ibuprofen and naproxen sodium, may help to decrease flow. If the hemoglobin is 10-12 g/dL, both observation and hormonal therapy are acceptable alternatives, as long as iron supplementation with 60 mg elemental iron per day is given. If hormonal therapy is decided on as the treatment choice, the possible regimens are the same as those for moderate anovulatory uterine bleeding, discussed below in detail. Re-evaluation should be made at three months or sooner if the bleeding persists or becomes more severe.

**Moderate Anovulatory Uterine Bleeding: **These patients can also be managed on an outpatient basis. In addition to iron supplementation, hormonal therapy is necessary to stabilize endometrial proliferation and shedding. There is no consensus on whether to treat with COCs or progestin-only regimens (40). In adolescents with moderate anemia who are actively bleeding, COCs are a better choice, as estrogen improves hemostasis (39). Monophasic COCs, containing at least 30 mcg of ethinyl E2, are preferred to prevent breakthrough bleeding. We recommend taking one pill every 8-12 hours until the bleeding stops, then to continue with one pill per day for a total of at least 21 days. If bleeding starts again dosing may be increased to twice a day for a total 21 days. 4-8 mg of ondansetron can be given if nausea occurs with high doses of E2 (30). At the end of 21 days, seven days of placebo or pause should be given. COCs treatment is continued for 3-6 months until the hemoglobin level reaches ≥12 g\dL. Different COCs regimens have been suggested in the literature (32,33,41). 

Progestin-only hormone therapy can be an alternative to COCs for adolescents with moderate anemia who are not currently bleeding or have a contraindication for estrogen therapy, such as arterial/venous thromboembolic disease, hepatic dysfunction, migraine with aura and/or estrogen dependent tumors (42,43). Progestin-only options are; micronized oral progesterone (200 mg/day), medroxyorigesterone (10 mg/day), norethindrone acetate (2.5-5 mg/day), depot-medroxyprogesterone acetate (DMPA) or a levonorgestrel-releasing intrauterine device. The last two options are not suitable for acute management but can be preferable for those who need contraception or cannot take pills. Micronized oral progesterone contains peanut oil and there must be caution for allergy. Also, there is no sufficient evidence to date to state that it is safer to use this progesterone than using synthetic progestin (44). However, micronized oral progesterone is chemically identical to endogenous progestin and this is more physiological. In some studies, it was also shown to have fewer side effects than synthetic progestin pills (45,46). Oral progestin is given for 12 days every month and bleeding occurs 2-7 days after cessation. If bleeding does not start within one week the patient should be re-evaluated.

**Severe Anovulatory Uterine Bleeding: **Patients with hemoglobin levels <7 g/dL and those with hemoglobin levels <10 g/dL but who have active heavy bleeding and hemodynamic instability (tachycardia, hypotension, orthostatic vital signs) must be hospitalized. They must be promptly evaluated in case blood transfusion is necessary. Patients with hemoglobin levels of 8-10 g/dL with parents who can reliably be contacted by telephone can be followed on an ambulatory basis (47). All patients with severe anemia due to menstrual bleeding must be assessed for bleeding disorders. Supplementation of 60-120 mg elemental iron must be started as soon as the patient is stable enough to take oral pills. 

Hormone therapy recommended for patients with hemoglobin levels of 8-10 g/dL consists of monophasic COCs containing 30-50 mcg ethinyl E2, given once every six hours for 2-4 days, followed by the same dose given every eight hours for three days and then every 12 hours for the next 14 days. Additional anti-emetic treatment may be necessary. For patients with hemoglobin levels <7 g/dL or <10 g/dL with heavy bleeding, COCs are given every four hours until bleeding slows down, followed with one pill every six hours for 2-3 days, every eight hours for three days and then every 12 hours for 2 weeks and continue with one pill a day until a hemoglobin level of ≥10 g/dL is reached and at least for a total of 21 days. When the hemoglobin level exceeds 10 g/dL, COCs are used in a cyclic pattern for three to six months until a hemoglobin level of ≥12 g/dL is attained (47).

If bleeding continues heavily after 24-hour administration of COCs or the patient is unable to take oral pills, 25 mg IV conjugated estrogen is given every 4-6 hours up to 2-3 times until the bleeding lessens. Then treatment is continued with oral COCs as described above. Physicians should remember the increased risk for thromboembolism with this therapy (48). Progestin-only therapies may be an option for patients who have a contraindication for COCs. For acute management, oral progestins are better than either DMPA or a levonorgestrel-releasing uterine device.

There may be a need for hemostatic agents such as tranexamic acid, aminocaproic acid and desmopressin, if bleeding exceeds 24 hours despite high dose COCs or there is a known platelet dysfunction. Tranexamic acid 3.9-4 g/day in three doses for 4-5 days is an effective treatment for HMB and is more effective than placebo (49). Although there is no evidence for increased incidence of thrombotic events associated with tranexamic acid, having a history of or active thromboembolic disease or an intrinsic risk for thrombosis are contraindications for tranexamic acid use. Concomitant usage of COCs increase the risk of thrombosis (49).

If hormonal and hemostatic treatment fail to lessen bleeding in 24-36 hours, examination under anesthesia, endometrial sampling and therapeutic curettage may be necessary (47,50).

## Follow-up

If irregular menses or HMB persists under hormone therapy for three months or recurs after cessation of therapy, the patient should be assessed for possible problems of hypothalamic-pituitary-ovarian axis, PCOS and structural causes. Adolescents with a history of untreated anovulatory cycles for 2-3 years should be evaluated by endometrial biopsy, as there is an increased risk for endometrial carcinoma in such patients (32).

## Experience of a Single Center

We evaluated the data of 22 patients with HMB referred to our adolescent outpatient clinic within a time period of 18 months. The mean age (range) of the patients was 13.9 years (11.3-16.9 years) and of menarche was 12.2 years (10.5-14.0 years) respectively, which is similar to the mean age of menarche in healthy Turkish girls. Half of the patients had been having heavy bleeding at each menses since menarche. Among those whose HMB began after menarche, the longest period between menarche and initiation of heavy bleeding was 3.3 years. Three of these patients had been given erythrocyte transfusions due to the degree of HMB prior to their admission to our hospital. The severity of bleeding was assessed as mild in five patients, moderate in three and severe in 14 patients. Seven of the severe patients had hemoglobin levels <8 g/dL. None of these patients were found to have platelet dysfunction or structural problems. One patient was diagnosed with hypothyroidism. Pelvic ultrasonography was compatible with PCOS in five patients. vWF antigen and activity was normal in all of the 18 patients who were assessed for vWF abnormalities. 

All patients were given oral iron supplementation. Treatment with a monophasic COC containing 0.15 mg desogestrel and 30 mcg ethinyl E2 was initiated in all patients with moderate and severe anemia, in accordance with the guidelines given above, except for two patients who were treated with oral tranexamic acid initially and then switched to COCs because of recurrent bleeding. Among patients with mild anemia, two patients were also treated with COCs, one with a two years history of irregularity predicting a lengthened anovulatory phase and one with endometrial hyperplasia. Two patients with a hemoglobin level of 5.8 g/dL and 5.9 g/dL required erythrocyte transfusions. Treatment with iron supplementation and/or COCs was successful in all patients over the short term (Table 4).

Thus, AUB is one of the major problems of adolescent gynecology and anovulatory HMB is the commonest presentation of AUB. Anovulatory cycles are generally physiologic and resolve spontaneously in most adolescents as the hypothalamic-pituitary-ovarian axis matures. Additionally, HMB may be due to coagulopathy and each adolescent with HMB should be questioned for bleeding disorders. Once hemodynamic stability is controlled and provided, the patient must be evaluated for severity of anemia and possible causes of HMB. Severity of anemia and its underlying cause determines the treatment. Beside iron supplementation, COCs, progestin only drugs, intravenous estrogen and\or hemostatic agents can be used for treatment. After treatment is stopped the patient should be followed for persisting anovulation.

## Figures and Tables

**Table 1:**
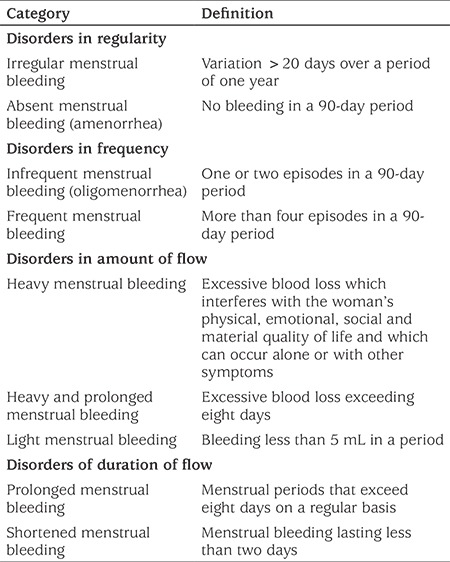
Abnormal uterine bleeding-The International Federation of Gynecology and Obstetrics recommendations for menstrual terminology

**Table 2:**
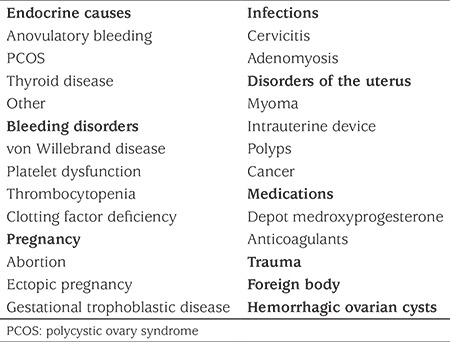
Differential diagnosis of heavy menstrual bleeding in adolescents

**Table 3:**
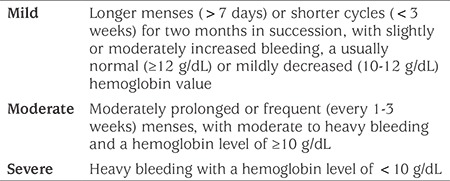
Severity classification

**Table 4:**
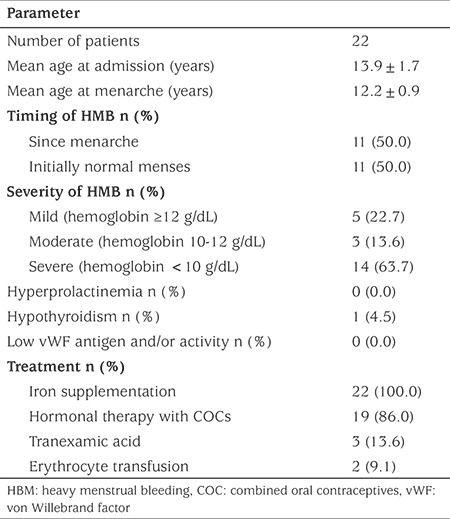
Clinical characteristics of our patients with heavy menstrual bleeding and their treatment
